# Whole-genome characterization and evolutionary analysis of a novel duck reovirus isolated from *Cairina moschata* in Vietnam

**DOI:** 10.14202/vetworld.2025.4172-4183

**Published:** 2025-12-31

**Authors:** Phan Nhan, Nguyen Tran Phuoc Chien, Nguyen Trong Ngu, Truong Quynh Nhu, Nguyen Phuc Khanh

**Affiliations:** 1 Interdisciplinary Graduate Program in Veterinary Therapeutics and Pathology, Faculty of Veterinary Medicine, College of Agriculture, Can Tho University, Can Tho, Vietnam; 2 Faculty of Applied Biology, Tay Do University, Can Tho, Vietnam; 3 Faculty of Veterinary Medicine, College of Agriculture, Can Tho University, Can Tho, Vietnam; 4 Faculty of Animal Sciences, College of Agriculture, Can Tho University, Can Tho, Vietnam

**Keywords:** amino acid variability, *Cairina moschata*, cross-border dissemination, genomic surveillance, novel duck reovirus, *Orthoreovirus*, phylogenetic analysis, shannon entropy whole-genome sequencing

## Abstract

**Background and Aim:**

Novel duck reovirus (NDRV) is an emerging *Orthoreovirus* infecting domestic waterfowl, particularly *Cairina moschata* (Muscovy duck), and is associated with arthritis, immunosuppression, and economic losses. Despite its circulation in Southeast Asia, whole-genome data from Vietnam are lacking, restricting molecular surveillance and regional vaccine development. This study aimed to sequence and characterize the complete genome of a Vietnamese NDRV isolate and evaluate its evolutionary relationship with global strains, with particular emphasis on lineage-specific mutations and entropy-based variability.

**Materials and Methods:**

Liver and spleen tissues from diseased *C. moschata* ducklings were used for virus isolation in specific-pathogen-free embryos. Viral RNA was extracted and subjected to whole-genome sequencing using the Illumina NovaSeq 6000 platform. Genome assembly, annotation, and quality assessment were performed through metaSPAdes, Ragtag, Prokka, and Geneious. Phylogenetic trees for all 10 segments were reconstructed using maximum-likelihood methods, and amino acid (aa) variability was quantified using Shannon entropy.

**Results:**

The Vietnamese isolate CTU/NDRV/TG.2024 possessed a 10-segment genome of 23,423 bp, with conserved terminal untranslated regions and canonical *Orthoreovirus* gene organization. Sequencing generated ~43.1 million paired-end reads with 100% genome coverage and a mean depth of 177×. The S1 segment was bicistronic, encoding P10, P18, and σC. Phylogenetic analyses of all segments consistently clustered the isolate within the NDRV lineage, closely related to Chinese isolates (2011–2023), notably J18 and SD-12, indicating recent regional dissemination. Aa identity across segments ranged from 89.2% to 99.6%, with the highest variability observed in S1 (particularly σC) and S4. High-entropy sites were concentrated within σC (positions 88N, 132A, 149A, 152Q), suggesting immune-driven antigenic drift. A unique substitution, L3-1248A, exhibited maximum entropy (*H*(x)=0.93), indicating a potential regional molecular signature. Complete genome sequences were deposited in GenBank (PV034361–PV034370).

**Conclusion:**

This study provides the first complete NDRV genome from Vietnam, revealing a close relationship to contemporary Chinese strains and highlighting mutation hotspots linked to immune-evasion and host-adaptation. These findings underscore the need for continued genomic surveillance and support the development of regionally appropriate vaccines for Southeast Asian waterfowl populations.

## INTRODUCTION

Novel duck reovirus (NDRV), an emerging pathogen within the genus *Orthoreovirus*, has become a major etiological agent responsible for morbidity and economic losses in domestic waterfowl, particularly *Cairina moschata* (Muscovy duck) and *Anas platyrhynchos domesticus* (Pekin duck). Since its initial detection in China in the late 2000s, NDRV has been associated with diverse clinical manifestations, including splenomegaly, hepatic necrosis, and, most prominently, viral arthritis and tenosynovitis, a syndrome that differs markedly from infections caused by classical avian reovirus (ARV) and Muscovy duck reovirus (MDRV) [1–3]. The virus possesses a segmented double-stranded RNA (dsRNA) genome consisting of 10 segments, a characteristic that facilitates frequent genetic reassortment and accelerates viral evolution. This genomic architecture contributes to the emergence of variants with modified virulence, expanded host range, and enhanced immune-evasion capacity [4, 5].

In Southeast Asia, where duck production constitutes an essential cultural and economic component, the spread of virulent NDRV strains poses significant risks to animal health, food security, and regional trade. Despite these threats, Vietnam remains substantially underrepresented in global genomic surveillance efforts. The scarcity of complete NDRV genome sequences hampers progress in molecular epidemiology, early detection, and the development of regionally optimized vaccines. Advances in next-generation sequencing (NGS) technologies have transformed RNA virus research, enabling comprehensive genome characterization and high-resolution phylogenetic analysis [6, 7]. The application of NGS to NDRV facilitates identification of lineage-defining mutations, host-adaptation signatures, and antigenic drift patterns that may affect diagnostic accuracy and vaccine performance [8, 9].

Although NDRV is increasingly recognized as a major pathogen in waterfowl across Asia, critical gaps remain in understanding its molecular epidemiology and evolutionary dynamics in Southeast Asia. Numerous complete NDRV genomes have been reported from China; however, Vietnam, despite its extensive *C. moschata* and *A. platyrhynchos domesticus* production systems, remains critically underrepresented in global genomic repositories. Existing Vietnamese data consist primarily of partial S1-segment sequences, limiting the ability to trace viral origins, detect reassortment, monitor antigenic drift, or assess cross-border dissemination driven by regional trade and wild bird migration [10]. Without full genomes, it is not possible to identify lineage-specific mutations, determine amino acid (aa) sites under selective pressure, or compare Vietnamese isolates with contemporary lineages circulating in China or neighboring countries. This deficiency restricts sensitive diagnostic development, complicates vaccine formulation, and weakens early-warning surveillance frameworks. Therefore, comprehensive whole-genome characterization of NDRV circulating in Vietnam is urgently needed to close this critical knowledge gap and strengthen regional disease-control strategies.

This study aimed to generate and analyze the first complete genome of an NDRV strain isolated from diseased C. moschata in Vietnam, thereby addressing the lack of regional genomic data. Specifically, the objectives were to: (i) sequence the full 10-segment dsRNA genome using NGS technologies; (ii) annotate genomic architecture and compare each segment with representative global NDRV isolates; (iii) construct maximum-likelihood (ML) phylogenies to determine the evolutionary position of the Vietnamese isolate relative to strains circulating in China and other countries; and (iv) identify lineage-specific aa substitutions and high-entropy regions suggestive of host-adaptation, immune escape, or regional divergence. Through this integrated genomic and phylogenetic approach, the study sought to create a foundational dataset for Vietnam, strengthen molecular surveillance systems, and support the development of diagnostics and vaccines tailored to Southeast Asian waterfowl populations.

## MATERIALS AND METHODS

### Ethical approval

All experimental procedures involving animals were reviewed and approved by the Institutional Animal Care and Use Committee / Animal Ethics Committee of Can Tho University, Vietnam (Approval No. CTU-AEC24030; Approval date: December 26, 2023). The study was conducted in strict accordance with the institutional animal welfare regulations, the national guidelines for the care and use of animals for scientific purposes in Vietnam, and internationally recognized principles for ethical animal research.

Field samples (liver and spleen tissues) were collected exclusively from naturally deceased *C. moschata* originating from disease-affected flocks. No animals were euthanized or experimentally infected for the purpose of this study, thereby minimizing animal use and distress.

Virus isolation was performed using specific-pathogen-free (SPF) embryonated duck eggs, following approved biosafety and animal welfare protocols. Embryos were monitored daily, and all procedures were carried out by trained personnel to ensure minimal suffering and adherence to humane endpoints.

The study complied with the principles of Replacement, Reduction, and Refinement (3Rs) by relying on field-derived samples, limiting the number of embryos used, and applying optimized laboratory protocols to reduce unnecessary repetition. All experimental activities were performed under biosafety-appropriate laboratory conditions, and no procedures involved endangered or protected species.

### Study period and location

The study was conducted from December 2023 to March 2024. Liver and spleen samples were collected from deceased *C. moschata* presenting with arthritis in Tien Giang Province, Mekong Delta, Vietnam, and processed for virus isolation, RNA extraction, and whole-genome sequencing (WGS) at the Faculty of Veterinary Medicine, Can Tho University.

The affected flock consisted of *C. moschata* aged 6–8 weeks, with estimated morbidity of 25%–30% and mortality of 5%–10%. Samples were collected only from naturally deceased birds. Metadata, including GPS coordinates (10.4030821, 106.6344997), clinical signs, and collection date, were deposited under BioSample SAMN53045226.

### Isolation and propagation of NDRV

Liver and spleen samples were aseptically collected from deceased *C. moschata* ducklings of the same flock showing arthritis. GenBank metadata (BioSample SAMN53045226; BioProject PRJNA1355538) were used to confirm sample origin.

Tissues were homogenized in sterile phosphate-buffered saline (pH 7.4) containing penicillin (100 IU/mL) and streptomycin (100 μg/mL), followed by centrifugation at 8,000 × g for 10 min at 4°C. Supernatants were clarified and filtered through 0.22 μm syringe filters.

Filtered supernatant (0.2 mL) was inoculated into the allantoic cavity of 10-day-old SPF embryonated duck eggs (n = 5). Eggs were incubated at 37°C and monitored. Allantoic fluid was harvested at 96 h post-inoculation or upon embryo death. Uninfected SPF embryos served as negative controls.

P1 allantoic fluid was used for sequencing to minimize culture-induced mutations. Mycoplasma and bacterial contamination were excluded through filtration and culture. Viral replication was confirmed using reverse transcription polymerase chain reaction targeting the S1 region.

### RNA extraction and complementary DNA (cDNA) synthesis

Total RNA was extracted from infected allantoic fluid using the NEXprep RNA Extraction Kit (Genes Laboratories Co., Ltd., South Korea). Samples with OD260/OD280 ≥ 2.0 were retained.

Approximately 1 μg RNA was reverse-transcribed using SensiFAST cDNA Synthesis Mix (Bioline, UK) in a 20 μL reaction containing random hexamers (200 ng), 5× TransAmp Buffer (4 μL), RNase-free water (7 μL), and reverse transcriptase (1 μL). Reaction conditions were: 25°C for 10 min → 48°C for 15 min → 85°C for 5 min.

RNA integrity number equivalent values (7.3–8.1) were determined by fragment analysis. RNA concentration was measured using Qubit fluorometry. Only samples meeting RNA ≥ 90 ng and OD260/OD280 ≥ 2.0 were used.

### Amplification of the S1 gene

The S1 gene was amplified using primers described by Liu *et al*. [2]:

- Forward: 5′-CTTTCGGGAATCGTGGTC-3′
- Reverse: 5′-CTGGACTCAGGCAGCGTA-3′

Each PCR mixture (25 μL) contained cDNA template (6 μL), primers (500 nM each), DNase/RNase-free water (11 μL), 5× MyTaq Reaction Buffer (5 μL), and MyTaq DNA Polymerase (1 μL; 2.5 U).

Thermal cycling conditions: 95°C for 15 s → 35 cycles of 95°C for 15 s, 60°C for 15 s, 72°C for 30 s → final extension at 72°C for 3 min.

Products were electrophoresed on 1.5% agarose stained with GelGreend™ (ABT, Vietnam) and visualized under ultraviolet (UV) illumination.

### WGS and assembly

RNA was further purified using the QIAamp Viral RNA Mini Kit (Qiagen, USA) and eluted in 30 μL RNase-free water. Samples with RNA ≥ 90 ng and OD260/OD280 ≥ 2.0 were used.

Libraries were prepared using the xGen™ BroadRange RNA Library Preparation Kit (Integrated DNA Technologies, USA). Fragment distributions were evaluated using the Bioanalyzer (Agilent Technologies). Libraries meeting ≥ 2 ng/μL concentration and ≥ 1 GB sequencing output were sequenced on the Illumina NovaSeq 6000 (150 bp paired-end), yielding 43,077,671 paired-end reads.

Quality filtering was performed with Fastp (version 0.23.1; Q30 = 94.1%; https://github. com/Open Gene/ fastp). Host-read removal eliminated ≥ 91.9% *A. platyrhynchos* reads using FastQScreen (https://www. bioinformatics.babraham.ac.uk/projects/fastq_screen/) and BBMap repair.sh. (https://sourceforge.net/projects/

bbmap/). The final assembly had 100% genome coverage, 177× mean depth, and 49.8% guanine–cytosine (GC) content.

*De novo* assembly was performed with metaSPAdes (version 3.15.5; https://github.com/ablab/spades). Viral contigs were identified using Basic Local Alignment Search Tool (BLAST; https://blast.ncbi.nlm.nih.gov/Blast.cgi), scaffolded with Ragtag (version 2.1.0; https://github.com/malonge/RagTag), and evaluated with CheckV (version 1.0.1; https://bitbucket.org/berkeleylab/CheckV). Reference assignment used Fast Average Nucleotide Identity (FastANI, version 1.33; https://github.com/ParBLiSS/FastANI) and ReferenceSeeker (version 1.8.0; https://github.com/oschwengers/referenceseeker).

Genome annotation was conducted using Prokka (version 1.14.6; https://github.com/tseemann/prokka) with the viral database (RVDB; version 24; https://rvdb.dbi.udel.edu). Final polishing was completed in Geneious Prime (version 2023.0.1; https://www.geneious.com).

### Phylogenetic and aa analysis

Multiple sequence alignment was performed using MAFFT (version 7.505; https://mafft.cbrc.jp/ alignment/software/). ML phylogenies for segments L1–L3, M1–M3, and S1–S4 were constructed using MEGA (version 12.0; https://www.megasoftware.net/).

Best-fit evolutionary models were selected using Bayesian Information Criterion:

- GTR+G+I for L and M segments
- HKY+G for S segments

Nodes with < 70% bootstrap support were collapsed. Trees were visualized in FigTree (version 1.4.4; https://github.com/rambaut/figtree).

Open reading frames (ORFs) were identified using ORFfinder (https://www.ncbi.nlm.nih.gov/orffinder/). Amino acid alignments were produced using BioEdit (version 7.2.5; http://www.mbio.ncsu.edu/BioEdit/ bioedit.html). Shannon entropy *H*(x) was calculated using a sliding window of 3 aa to identify mutation hotspots.

## RESULTS

### CTU/NDRV/TG.2024 sequencing data

Sequencing of the NDRV strain CTU/NDRV/TG.2024 yielded a complete genome of 23,423 bp. A total of 43,077,671 paired-end reads were generated, resulting in an average sequencing depth of 177×, full nucleotide coverage (100%), and an overall GC content of 49.8%. Conserved 5′ and 3′ untranslated regions (UTR) motifs were identified across all segments, showing high similarity to the terminal sequences GCUUUU(U) at the 5′ end and UCAUC at the 3′ end. The 5′ UTR length ranged from 12 nt in L3 to 30 nt in S3, while the 3′ UTR varied from 32 nt in S1 to 101 nt in M2. The complete genomic annotation of the isolates is presented in Table 1.

**Table 1:** Genomic characteristics of CTU/NDRV/TG.2024, including segment length, terminal sequences, ORF organization, protein size, and predicted protein function.

Genomic segment	Acession numbers	Length (bp)	Length (bp) of the 5′-end ORF-3′	Sequence at the 5′–3′ end termini	ORF location		Protein size (aa)	Encoded protein

					Start	End		
L1	PV034361	3959	21-3882-56	GCUUUUU/UCAUC	22	3903	1293	λA (core shell)
L2	PV034369	3830	14-3780-36	GCUUUUU/UCAUC	15	3794	1271	λB (core RdRp)
L3	PV034370	3907	12-3858-37	GCUUUUU/UCAUC	13	3870	1285	λC (core turret)
M1	PV034362	2284	13-2199-72	GCUUUUU/UCAUC	14	2212	736	μA (core NTPase)
M2	PV034363	2158	29-2028-101	GCUUUUU/UCAUC	30	2057	675	μB (outer shell)
M3	PV034364	1996	24-1908-64	GCUUUUU/UCAUC	25	1932	635	μNS (NS factory)
S1	PV034365	1568	19-1517-32	GCUUUUU/UCAUC	20	313	97	P10 (Ns-FAST)
					273	761	162	P18 (NS other)
					571	1536	321	σC (outer fiber)
S2	PV034366	1324	15-1251-58	GCUUUUU/UCAUC	16	1266	416	σA (outer clamp)
S3	PV034367	1202	30-1104-68	GCUUUUU/UCAUC	31	1134	367	σB (outer clamp)
S4	PV034368	1195	27-1104-64	GCUUUUU/UCAUC	28	1131	367	σNS (NS RNAb)

NDRV = Novel duck reovirus, ORF = Open reading frame, bp = Base pair, aa = Amino acid, RdRp = RNA-dependent RNA, NS = Non-structural, NTPase = Nucleoside triphosphatase, FAST = Fusion-associated small transmembrane protein, RNAb = RNA-binding

Genome cloning, sequencing, and BLAST verification confirmed CTU/NDRV/TG.2024 as NDRV. The complete genome was deposited in GenBank under accession numbers PV034361–PV034370. Consistent with the genus *Orthoreovirus*, the genome consisted of 10 dsRNA segments: L1 (3959 bp), L2 (3830 bp), L3 (3907 bp), M1 (2284 bp), M2 (2158 bp), M3 (1996 bp), S1 (1568 bp), S2 (1324 bp), S3 (1202 bp), and S4 (1195 bp).

ORF prediction demonstrated that the S1 segment was bicistronic, encoding P10 (20–313 bp), P18 (273–761 bp), and σC (571–1536 bp). All remaining segments contained a single ORF encoding λA, λB, λC, μA, μB, μNS, σA, σB, and σNS, consistent with established *Orthoreovirus* genome architecture.

### Genomic analysis

#### L-Class segments (L1–L3)

**Figure 1:**
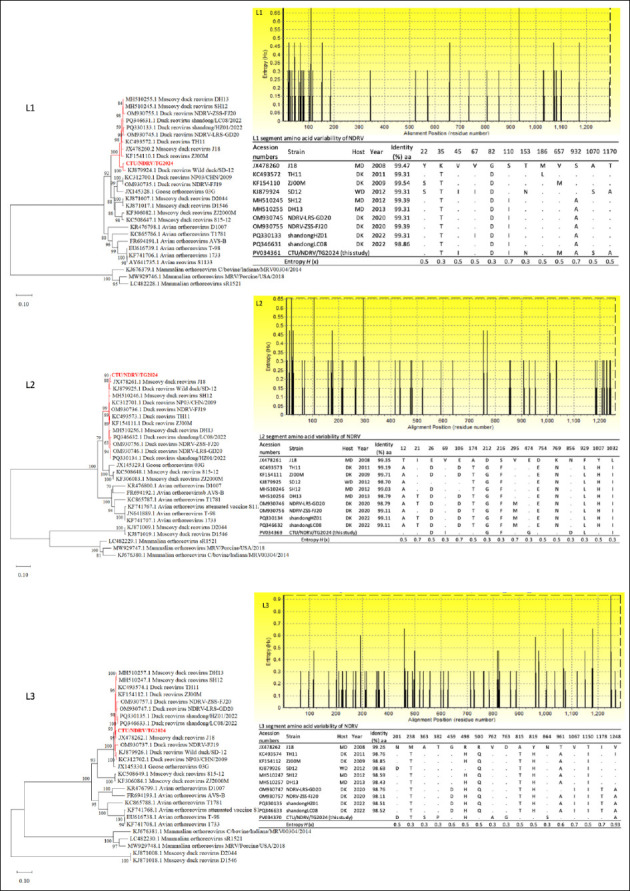
Phylogenetic relationships and amino acid (aa) variability of the Novel duck reovirus (NDRV) L1, L2, and L3 segments. Maximum-likelihood phylogenetic trees were constructed based on the complete nucleotide sequences of the L1, L2, and L3 segments from the CTU/NDRV/TG.2024 Vietnamese isolate (in red) and reference *Orthoreovirus* strains retrieved from GenBank. The bootstrap replicates, and the threshold for displaying values (>70%) from 1,000 replicates, are shown at the nodes. Corresponding aa variability plots depict Shannon entropy (*H*(x)) across aligned sequences, with higher peaks indicating greater variability. Tables summarize aa sequence identity (%) and substitutions at variable positions relative to the reference strain J18.

Phylogenetic reconstruction of the L1 segment placed CTU/NDRV/TG.2024 within the same clade as the Chinese strain SD-12 (isolated from a wild duck in 2012) and clustered it with NDRV isolates reported in China from 2011 to 2023, including DH13, SH12, TH11, ZJ00M, J18, NDRV-LRS-GD20, NDRV-ZSS-FJ20, Shandong/ HZ01/2022, and Shandong/LC08/2022 (Figure 1). Similar clustering was observed for L2, where CTU/ NDRV/ TG.2024 grouped with J18 and SD-12 to form a strongly supported monophyletic cluster (bootstrap = 99%). L3 phylogeny also positioned CTU/NDRV/TG.2024 closely with J18 and other isolates from ducks, wild birds, and *C. moschata* collected between 2011 and 2023 (bootstrap = 93%).

Across all L-class segments, NDRV remained clearly separated from MDRV. Two French MDRV isolates (D2044 and D1546) showed the greatest divergence from other *Orthoreovirus* lineages. These findings confirm that CTU/NDRV/TG.2024 is evolutionarily close to contemporary Chinese NDRV strains and may represent a lineage currently circulating in Southeast Asia.

Amino acid identity between CTU/NDRV/TG.2024 and 10 representative NDRV strains ranged from 98.86%–99.54% (L1), 98.52%–99.35% (L2), and 98.11%–99.26% (L3). Segment-specific substitutions included:

- L1: Nine substitutions (35T, 45I, 82D, 101I, 153N, 657M, 932A, 1070S, 1170A), with high-entropy sites at positions 110 (S→I) and 932 (S→A; entropy = 0.7).
- L2: Eight substitutions (26D, 69I, 212G, 216F, 474G, 856D, 929L, 1032I).
- L3: Ten substitutions (201D, 238T, 363S, 382P, 489H, 762A, 763G, 864S, 1248A), with 1248A showing the highest entropy (0.93).

#### M-class segments (M1–M3)

Phylogenetic analysis of the M-class segments confirmed clear distinctions among ARV, MDRV, and NDRV lineages. CTU/NDRV/TG.2024 consistently grouped within the NDRV cluster. The M1 phylogeny showed a close relationship with Chinese strains such as NDRV-LRS-GD20, NDRV-ZSS-FJ20, Shandong/HZ01/2022, DH13, SH12, TH11, ZJ00M, NDRV-FJ19, NP03/CHN/2009, and J18, although CTU/NDRV/TG.2024 formed a distinct sub-branch. Both M2 and M3 segments clustered closely with recent Chinese isolates (2011–2023), reflecting substantial evolutionary conservation.

**Figure 2:**
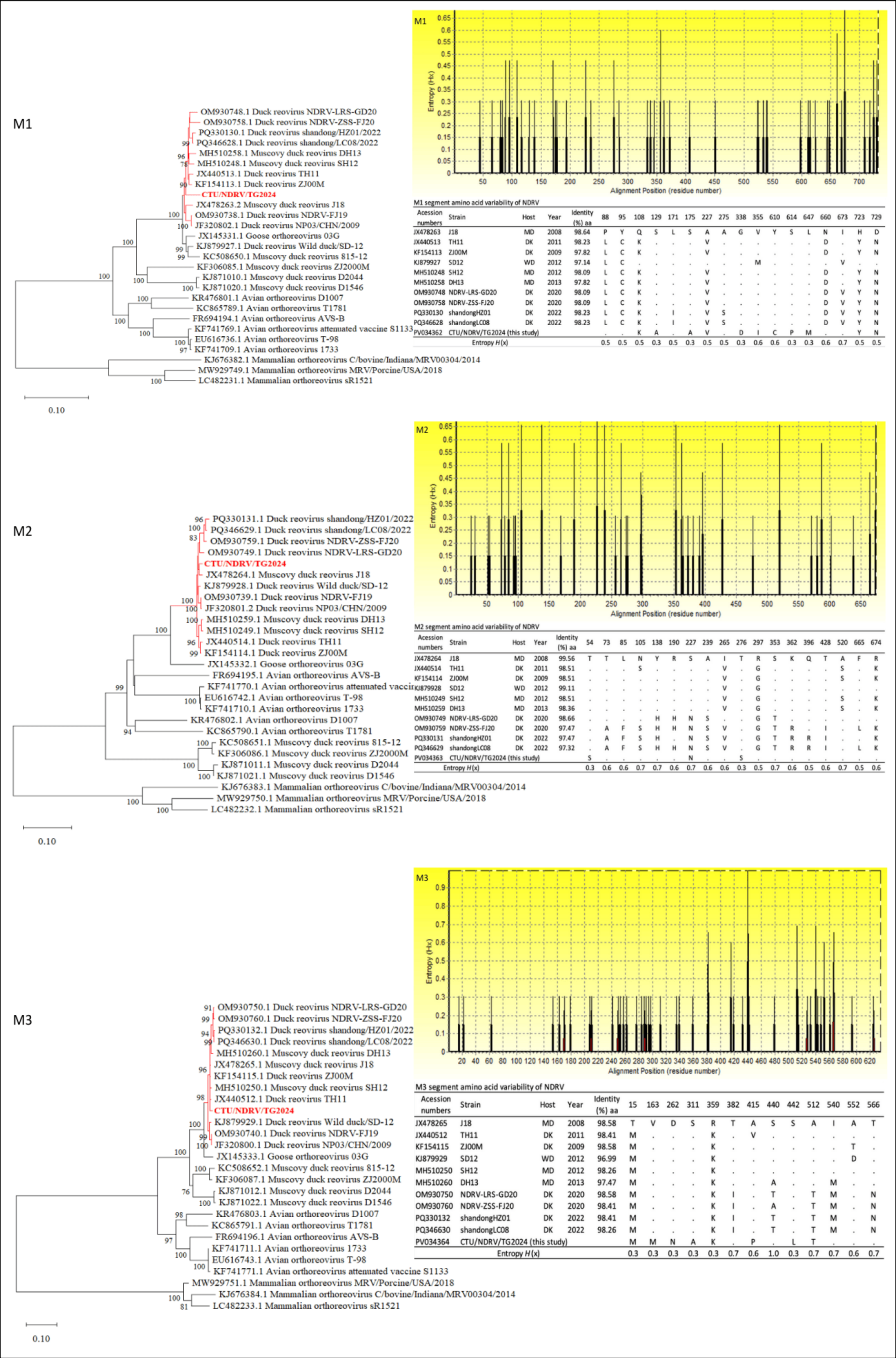
Phylogenetic relationships and amino acid (aa) variability of the Novel duck reovirus (NDRV) M1, M2, and M3 segments. Maximum-likelihood phylogenetic trees were constructed based on complete nucleotide sequences of M1, M2, and M3 segments from the Vietnamese isolate CTU/NDRV/TG.2024 (in red) and reference *Orthoreovirus* strains retrieved from GenBank. The bootstrap replicates, and the threshold for displaying values (>70%) from 1,000 replicates, are shown at the nodes. Corresponding aa variability plots depict Shannon entropy (*H*(x)) across aligned sequences, with higher peaks indicating greater variability. Tables summarize aa sequence identity (%) and substitutions at variable positions relative to the reference strain J18.

Amino acid identity ranged from 97.82%–98.64% (M1), 97.32%–99.55% (M2), and 98.26%–99.58% (M3), with M3 being the most conserved (Figure 2).

Key substitutions included:

- M1: Eleven substitutions (108K, 129A, 175A, 227V, 338D, 355I, 610C, 614P, 647M, 723Y, 729N), with entropy hotspots at positions 660 (0.6) and 673 (0.7).
- M2: Three substitutions (54S, 227N, 276S) and high-entropy sites (0.6–0.7) across residues 85–239, corresponding to domains involved in receptor interaction and outer capsid structure.
- M3: Substitutions 415P, 512T, and 440S (entropy 0.6–1.0), along with Vietnamese-specific mutations (163M, 262N, 311A, 442L) potentially influencing viral polymerase function or virion stability.

#### S-class segments (S1–S4)

**Figure 3:**
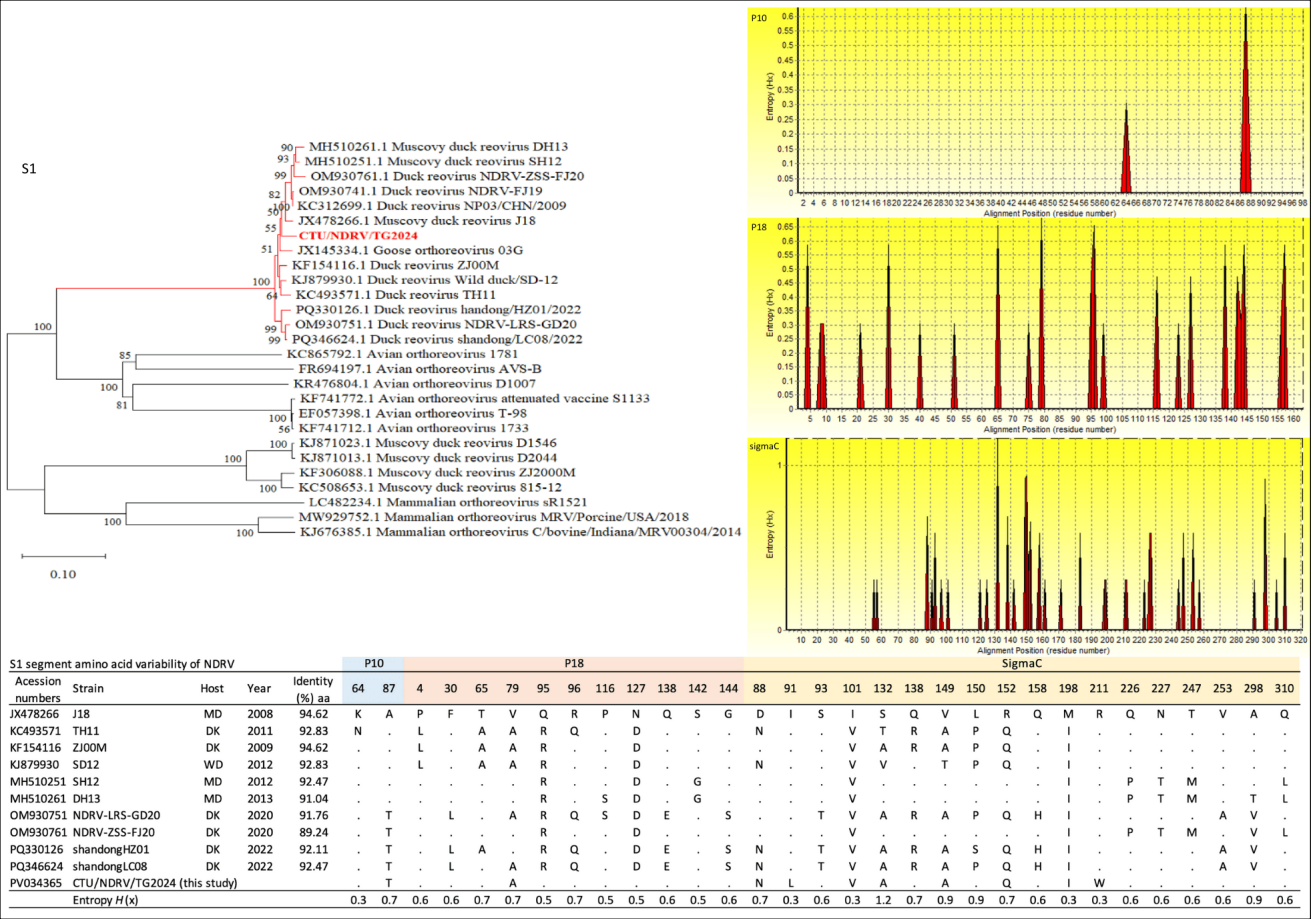
Phylogenetic relationships and amino acid (aa) variability of the Novel duck reovirus (NDRV) S1 segments. Maximum-likelihood phylogenetic trees were constructed based on complete nucleotide sequences of S1 segments from the Vietnamese isolate CTU/NDRV/TG.2024 (in red) and reference *Orthoreovirus* strains retrieved from GenBank. The bootstrap replicates, and the threshold for displaying values (>70%) from 1,000 replicates, are shown at the nodes. Corresponding aa variability plots depict Shannon entropy (*H*(x)) across aligned sequences, with higher peaks indicating greater variability. Tables summarize aa sequence identity (%) and substitutions at variable positions relative to the reference strain J18.

**Figure 4:**
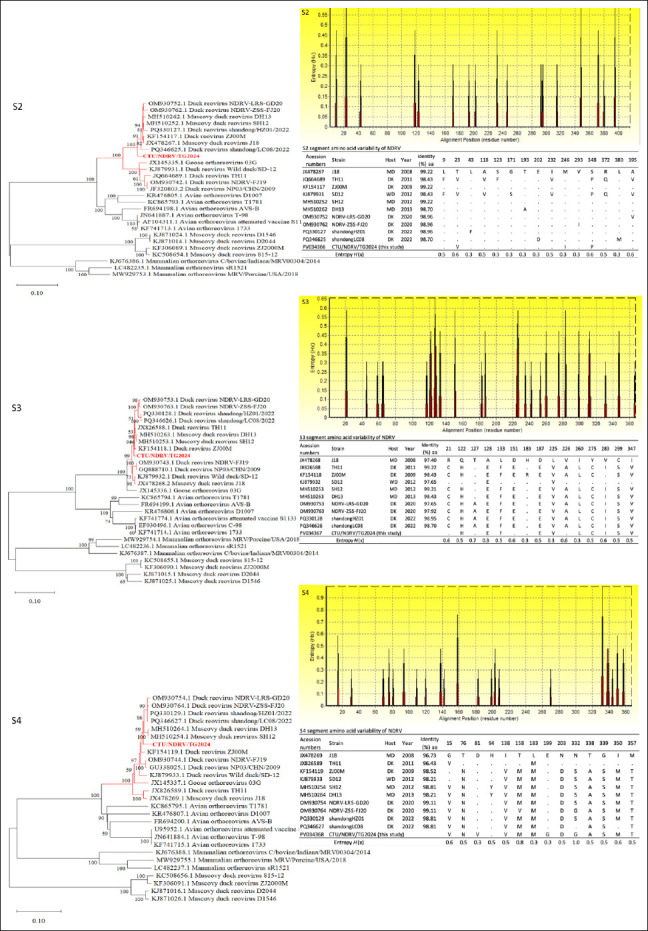
Phylogenetic relationships and amino acid (aa) variability of the Novel duck reovirus (NDRV) S2, S3, and S4 segments. Maximum-likelihood phylogenetic trees were constructed based on the complete nucleotide sequences of the S2, S3, and S4 segments from the Vietnamese isolate CTU/NDRV/TG.2024 (in red) and the reference *Orthoreovirus* strains retrieved from GenBank. The bootstrap replicates, and the threshold for displaying values (>70%) from 1,000 replicates, are shown at the nodes. Corresponding aa variability plots depict Shannon entropy (*H*(x)) across aligned sequences, with higher peaks indicating greater variability. Tables summarize aa sequence identity (%) and substitutions at variable positions relative to the reference strain J18.

The S1 phylogeny placed CTU/NDRV/TG.2024 within a well-supported NDRV subcluster (2011–2023), closely related to J18, NP03/CHN/2009, NDRV-FJ19, NDRV-ZSS-FJ, DH13, and SH12 (Figure 3). The S2 segment formed a monophyletic group with J18, ZJ00M, Shandong/HZ01/2022, and NDRV-LRS-GD20. The S3 segment also clustered with recent Chinese isolates, while S4 showed mild divergence characteristic of Southeast Asian variants but remained tightly grouped with contemporary Chinese strains (Figure 4).

Amino acid identity ranged from 89.25%–94.62% (S1), 97.38%–99.22% (S2), 96.43%–99.22% (S3), and 96.43%–99.11% (S4) (Figures 3 and 4). Variability in S1 and S4 is consistent with selective immune pressure.

Key mutations included:

- S1–P10: One substitution (87A→T; entropy 0.7).
- S1–P18: One substitution (79A; entropy 0.7).
- S1–σC: Multiple substitutions (88N, 91L, 101V, 132A, 149A, 152Q, 198I, 211W), with high-entropy sites at positions 88 (0.7), 132 (1.2), 149 (0.9), and 152 (0.7).
- S2: Substitutions 23V, 246I, and 348P (entropy ≤ 0.6).
- S3: Ten substitutions (122H, 128E, 133F, 187E, 225V, 260L, 275C, 283I, 299S, 347V), with entropy values between 0.5 and 0.7.
- S4: Multiple substitutions (15V, 76V, 81V, 138V, 158M, 183M, 193G, 203D, 332G, 338A, 339S, 350M, 357T), with 332G showing maximum entropy (1.0), indicating strong selective pressure.

Overall, S1—particularly σC—and S4 displayed the highest mutation densities, supporting immune-driven antigenic drift and regional viral adaptation.

## DISCUSSION

### Genomic characterization of CTU/NDRV/TG.2024

NGS was used to determine and characterize the complete genome of the Vietnamese NDRV strain CTU/NDRV/TG.2024. The assembled genome measured 23,423 bp and comprised 10 dsRNA segments, consistent with the genomic structure typical of the genus *Orthoreovirus* [1, 2, 7, 10, 11]. This analysis provides essential insights into the genetic architecture, molecular features, and evolutionary dynamics of the strain.

This study reports the first complete NDRV genome from Vietnam, thereby addressing a significant geographic gap in Southeast Asian molecular surveillance of ARV. Multiple unique aa substitutions and high-entropy hotspots were identified, suggesting ongoing local viral adaptation. The conserved terminal UTRs and ORF structure, particularly the bicistronic S1 segment encoding P10, P18, and σC, were consistent with previously described NDRV and other avian *Orthoreovirus* genomes [6, 12, 13].

### Phylogenetic relationships and evolutionary positioning

Across L-, M-, and S-class segments, phylogenetic analyses demonstrated a close evolutionary relationship between CTU/NDRV/TG.2024 and Chinese NDRV isolates reported between 2011 and 2023 [3, 7, 14–16]. The Vietnamese isolate consistently clustered with strains such as J18, SD-12, NDRV-LRS-GD20, NDRV-ZSS-FJ20, Shandong/HZ01/2022, and Shandong/LC08/2022. These findings suggest shared ancestry and ongoing transboundary circulation of NDRV lineages among domestic and wild waterfowl populations in Southeast Asia and China [3, 7, 15, 17, 18].

Movement of live ducks, cross-border farming networks linking Vietnam with Laos, Cambodia, and southern China, and migration of wild birds likely contribute to this dissemination, aligning with One Health perspectives on regional pathogen transmission. Distinct phylogenetic clustering of NDRV, MDRV, and ARV indicates host-associated divergence, although NDRV remains capable of infecting various avian species, including chickens and geese [3, 19, 20]. Greater divergence was observed in S1 and S4 segments, suggesting region-specific adaptation, whereas S2 and S3 remained highly conserved relative to Chinese reference strains [6, 21].

### Comparative aa diversity among genome segments

Comparative aa analyses revealed heterogeneous variability across genome segments. L-class (L1–L3) and M-class (M1–M3) segments exhibited generally high aa identity, with M3 being the most conserved [5, 11]. By contrast, S-class segments (S1–S4), notably S1, displayed markedly higher variability, consistent with immune selection pressures acting on proteins such as σC, σB, and μB, which mediate host cell attachment and immune recognition [5, 10, 11, 22, 23].

Several high-entropy substitutions were observed across segments. For example, in L1, positions 110 (S→I) and 932 (S→A) displayed notable variability, whereas L2 showed entropy peaks at sites 21I and 106E [24]. Similarly, M-class entropy hotspots included M1-355I and M1-610C, as well as M3 positions such as 415P and 512T. Additional Vietnamese-specific substitutions, 163M, 262N, 311A, and 442L, may influence polymerase function or virion stability [3, 5, 11, 19].

### Hypervariability and immune-driven selection in S-class segments

The S1–σC region exhibited the highest mutation density and entropy values, including substitutions at 88N, 91L, 101V, 132A, 149A, 152Q, 198I, and 211W, with entropy peaks exceeding 1.0 at several positions [6, 10, 12, 28]. σC functions as a key secondary outer capsid protein responsible for receptor–binding and induction of neutralizing antibodies [6, 8]. Consequently, hypervariability within σC contributes to antigenic divergence and may reduce the effectiveness of vaccine-induced immunity [3, 23].

In regions such as the Mekong Delta, where mobile-duck and smallholder production systems dominate, antigenic drift may exacerbate challenges in outbreak detection, immune escape, and disease management. The L3-1248A substitution (entropy = 0.93) is particularly noteworthy as a potential molecular signature of emerging Vietnamese NDRV variants.

Future functional studies, including neutralization assays, structural modeling, and reverse genetics, will be essential to determine whether mutations such as σC-132A, σC-149A, σC-152Q, and L3-1248A influence receptor affinity, antibody escape, replication efficiency, or virulence.

### Implications for viral evolution, disease–control, and surveillance

The collected data provide compelling evidence of rapid NDRV evolution, including the potential emergence of variants with altered virulence and host range [3, 15, 20, 21, 24, 25]. The segmented nature of the genome facilitates reassortment, a major driver of novel phenotype emergence [1, 3, 24]. Mutations affecting polymerase function, capsid stability, and receptor–binding regions emphasize the importance of vigilant molecular surveillance.

Previous research by Yang *et al*. [7] and Wang *et al*. [13] highlighted the need to integrate genomic monitoring with laboratory characterization to assess impacts on virulence, host-adaptation, and vaccine efficacy [13, 15, 19, 26].

### Public health and vaccine development considerations

Rapid viral evolution underscores the importance of updating diagnostic assays to detect emerging variants [3, 9, 26]. Antigenic diversity, particularly involving σC, poses notable challenges for vaccine development, highlighting the need for multivalent or broadly protective vaccine platforms [8, 9, 27, 28]. Continuous genomic monitoring at the domestic–wild waterfowl interface is vital for detecting cross-species transmission and optimizing biosecurity strategies. A limitation of this study is the analysis of a single isolate. Broader sampling across multiple geographic regions, waterfowl species, production systems, and timepoints is required to fully characterize NDRV diversity in Vietnam. High-entropy mutations identified here should be evaluated experimentally to determine their biological relevance.

## CONCLUSION

This study provides the first complete whole-genome characterization of a Vietnamese NDRV strain, CTU/NDRV/TG.2024, isolated from C. moschata exhibiting arthritis in the Mekong Delta. High-depth NGS revealed a 23,423 bp genome with conserved terminal UTRs and a canonical Orthoreovirus 10-segment structure. Phylogenetic analyses consistently placed CTU/NDRV/TG.2024 within the contemporary NDRV lineage circulating in China between 2011 and 2023, clustering particularly with isolates such as J18 and SD-12. Comparative genomic analysis identified uneven aa variability across the genome, with marked divergence in the S1–σC and S4 segments and a unique high-entropy L3–1248A substitution, suggesting ongoing regional adaptation, immune-driven selection, and the possible emergence of a localized viral signature in southern Vietnam.

The findings have important practical implications. The close genetic relatedness to Chinese NDRV strains implies active transboundary movement through duck trade networks or migratory waterfowl, underscoring the need for coordinated regional surveillance. The presence of high-variability sites in σC, a key antigenic protein, raises concerns regarding vaccine match and diagnostic accuracy, emphasizing the need for continuous genomic monitoring and evaluation of existing vaccine strains used across Southeast Asian waterfowl production systems.

A notable strength of this study is the generation of the first complete NDRV genome from Vietnam, filling a critical surveillance gap and providing foundational data for molecular epidemiology, vaccine development, and early-warning systems. However, the conclusions are limited by the analysis of a single isolate, which may not fully reflect the breadth of NDRV diversity circulating in the region. Broader sampling across multiple provinces, host species, and production systems will be essential to define lineage diversity, quantify reassortment events, and understand spatiotemporal viral evolution.

Future research should focus on functional characterization of high-entropy mutations, especially those within σC and polymerase-associated regions, to determine their impact on virulence, receptor–binding, immune escape, and vaccine protection. Integrating genomic surveillance with field epidemiology, serological monitoring, and experimental infection studies will further enhance regional control strategies.

In conclusion, this study demonstrates that Vietnamese NDRV exhibits close evolutionary links with contemporary Chinese lineages while harboring distinct mutations indicative of local adaptation. These insights underscore the urgency of sustained genomic surveillance, strengthened biosecurity, and updated vaccines to mitigate the emerging threat of NDRV in Southeast Asian duck production systems.

## DATA AVAILABILITY

Complete genome segments of strain CTU/NDRV/TG.2024 are available in GenBank (PV034361–PV034370). Raw sequencing reads will be deposited in the NCBI SRA under BioProject PRJNA1355538 and BioSample SAMN53045226

## AUTHORS’ CONTRIBUTIONS

PN: Conceptualization, methodology, formal analysis, investigation, data curation, and drafted and revised the manuscript. NTPC: Investigation and data curation. TQN: Validation and data curation. NTN: Formal analysis, data curation, drafted and revised the manuscript. NPK: Conceptualization and revised the manuscript. All authors have read and approved the final manuscript.
